# SOCIAL ISOLATION, LONELINESS, AND THE LIVING ARRANGEMENTS AND CONTEXTS OF OLDER ADULTS

**DOI:** 10.1093/geroni/igae098.0824

**Published:** 2024-12-31

**Authors:** Rebecca Mauldin, Lydia Li

**Affiliations:** Chair; Discussant

## Abstract

Many older adults and their caregivers are at risk for social isolation and loneliness, which carry substantial adverse effects for health and well-being. Researchers and practitioners recognize the need for tailored interventions to address the unique circumstances of individuals and groups experiencing social isolation and loneliness. One factor that should be considered is living arrangements (e.g., living alone or in residential care settings) or living contexts such as neighborhood environment. The papers presented in this symposium seek to advance research related to the role of living arrangements or contexts on the social isolation and loneliness of older adults. The first paper examines older adults living alone and without care partners with a scoping review related to their risks and vulnerabilities, the adverse consequences of this living situation; and related interventions. The second paper focuses on the social networks of older adults in an assisted living community and presents quasi-experimental findings of network changes associated with online nature-based group activity programming in assisted living. The third paper examines the role of perceived neighborhood disorder and social support in caregivers’ loneliness using data from the Health and Retirement Study to identify the association between neighborhood stressors, social support, and loneliness. The final paper presents findings from a mixed methods study examining the relationship between loneliness, living arrangements, and social support among caregivers of persons living with dementia. Overall, the symposium demonstrates ways in which living arrangements and contexts can be considered in research and practices with older adults and their caregivers.

